# Elevated number and density of macrophage-like cell as a novel inflammation biomarker in diabetic macular edema

**DOI:** 10.1038/s41598-023-32455-1

**Published:** 2023-03-31

**Authors:** Zongyi Wang, Haiyan An, Jiyang Tang, Enzhong Jin, Siying Li, Linqi Zhang, Lvzhen Huang, Jinfeng Qu

**Affiliations:** 1 Department of Ophthalmology, Eye Diseases and Optometry Institute, Peking University People’s Hospital, Beijing, 100044 China; 2 Beijing Key Laboratory of Diagnosis and Therapy of Retinal and Choroid Diseases, College of Optometry, Peking University Health Science Center, Beijing, China; 3 Department of Anesthesiology, Peking University People’s Hospital, Beijing, China

**Keywords:** Diseases, Eye diseases, Macular degeneration, Retinal diseases

## Abstract

To quantitatively analyze the number and density of macrophage-like cells (MLCs) at the vitreoretinal interface at macular region in diabetic retinopathy (DR) with and without diabetic macular edema (DME). This cross-sectional study involved 240 eyes of 146 treatment-naïve DR patients, including 151 eyes with DME. The number and density of MLCs were analyzed quantitatively using optical coherence tomography angiography (OCTA) and were compared between DME and non-DME eyes as well as proliferative DR (PDR) and non-PDR (NPDR) eyes. Correlation between MLCs density and vessel density of macular superficial capillary plexus (SCP) at macular region was evaluated. The number and density of macular MLCs were both elevated in DME group compared to non-DME group (all p < 0.001). The morphology of MLCs in DME eyes appeared larger and fuller. NPDR eyes had higher number and density of MLCs (p = 0.027 and 0.026), greater central macular thickness (CMT) (p = 0.002) and vessel density than PDR eyes in non-DME group but comparable to PDR eyes in DME group. The number and density of MLCs at macular region were significantly higher with larger and fuller morphology in DR patients with DME than those without DME. PDR eyes had fewer MLCs than NPDR eyes for DR eyes without DME.

## Introduction

With the increasing prevalence of diabetes worldwide, diabetic retinopathy (DR) is becoming one of the leading causes of visual impairment in working population in many countries^1^. As one of the visual-threatening causes in DR patients, DME may occur at any stage^2–4^.

Several studies have demonstrated that macrophage-like cells (MLCs), as a novel inflammation biomarker could be observed at the vitreoretinal interface using optical coherence tomography angiography (OCTA)^5–7^. Increased activation of microglia and hyalocytes was believed to be related to MLCs^3,8^. Microglia, as the resident immune cells in the retina and brain, are rapidly activated at the onset of retinal injury to facilitate antigen presentation and phagocytosis^5^. Microglia are mainly located in the inner and outer plexiform layer of retina, but can also be found in the ganglion cell layer, retinal nerve fiber layer and close to the inner limiting membrane (ILM)^9^. Hyalocytes are thought to be the resident macrophages of the vitreous with two distinct morphological and functional subpopulations^10^. The primary physiological role of hyalocytes is to maintain the transparency of the vitreous by scavenging cellular debris and secreting anti-angiogenic factors to inhibit blood vessel growth^11^.

Previous studies suggest that MLCs may play an important role in the occurrence and progression of retinal diseases^3,12^. OCT have been used to observe MLCs in retinal vein occlusion and diabetic retinopathy^5,7,13^. However, there has been no study compared MLCs changes in DR with and without DME. Since inflammatory reaction was believed to participated in the pathogenesis of DME, quantitative analysis of MLCs as an inflammatory biomarker in DME may help us to understand the mechanism of DR and DME better and may provide another novel predictor for the treatment response to different agent.

This study aimed to quantitatively analyze the number, density and morphology changes of MLCs at the vitreoretinal interface in DR patients with and without DME by using en face optical coherence tomography (OCT).

## Methods

### Subjects

This cross-sectional study enrolled the treatment-naïve diabetic retinopathy patients from January 2020 to April 2022 at the Eye Center of Peking University People’s Hospital, Beijing, China. The research adhered to the tenets of the Declaration of Helsinki and was approved by the Ethical Committee of Peking University People’s Hospital. The written informed consent was obtained from all participants. All included subjects underwent a comprehensive ophthalmic examination, including best-corrected visual acuity (BCVA), intraocular pressure, slit lamp examination, fundus examination, fluorescein fundus angiography (FFA) and OCTA. Subjects with following conditions were excluded: (1) history of intraocular surgery or ocular trauma (vitreoretinal retinal surgery, intravitreal injection, laser therapy); (2) presence or history of other eye diseases (age-related macular degeneration, glaucoma, uveitis etc.); (3) refractive error > 3 diopters (D); (4) corneal and lens pathologies that prevent a clear view of the fundus; (5) other serious systemic diseases (cancer, infectious disease, inherited metabolic diseases, mental disorder, severe hypertension, acute myocardial infarction, stroke); (6) poor OCTA image quality (scan quality < 6 or obvious motion artifact). The morphological patterns of DME were classified on the basis of OCT into three groups—diffuse retinal thickening (DRT), cystoid macular edema (CME), and serous retinal detachment (SRD). The leakage features of DME in FFA were categorized into focal leakage type, diffuse leakage type and mixed leakage type.

### OCTA images acquisition and processing

We used the high-density (HD) macular angiography 6 × 6 mm (350 × 350 scan, 661 × 662 pixels) scan protocol of Cirrus HD-OCT 5000 (Carl Zeiss Meditec Inc, Dublin, CA, USA) to acquire the macular images centered on the fovea. Central macular thickness (CMT), vessel densities of superficial capillary plexus (SCP) in the central, inner circle (3 mm radius), outer circle (6 mm radius) and overall macular region of ETDRS grid were recorded. The segmentation of each scan was checked and corrected manually if they deviated from the right position. In addition, we manually counted the total number of retinal hyperreflective foci (HRF) within scanned area and graded the integrity of ELM/EZ in the central subfield. Based on previous studies^14,15^, to avoid the misjudgment of hard exudates or microaneurysms, the following criteria were adopted for the selection of HRF: (1) well-circumscribed dots that were 10–40 μm in diameter; (2) reflectivity similar to the retinal nerve fiber layer; (3) no back-shadowing. The integrity of external limiting membrane (ELM)/ellipsoid zone (EZ) was graded on the basis of the visibility and continuity of the first and second hyperreflective layers of the most four outer layers and was categorized as intact (discernible and continuous), disrupted (partially visible) and absent (completely lost)^14,16^.

According to the descriptions of previous studies, adjusting a 3-mm slab above the ILM on macular region is effective in obtaining clear images of MLCs^5,7,13^. A custom slab from 0 to 3 μm above the ILM on macular regions was manually adjusted and the en face OCT images were exported for further processing. Then we imported these images into FIJI software, a distribution of the program ImageJ (National Institutes of Health, Bethesda, MD, USA) and used a semiautomated binarization protocol (including three general components: noise reduction to remove background irregularities and vessel artifacts, signal enhancement to improve cell identification, and binarization to extract discrete cell shapes) in a masked fashion to identify and isolate the MLCs on the extracted 3-mm slab^7^ (Supplementary File 1). The number and density of MLCs were qualified on the binarized images by using the analyze particles function of ImageJ. MLC density was defined as the number of cells divided by total image area (cells/mm^2^).

### Statistical analysis

Statistical analyses were performed using GraphPad Prism 9.1.0 software (GraphPad Software Inc., La Jolla, California USA). Demographic and outcome data were described by frequency for categorical variables or mean ± SD for continuous variables. Student’s t-test was used to compare differences of parametric data between groups. Pearson’s correlation coefficient was used to evaluate the correlation between MLC density, macular vessel density and central macular thickness. p < 0.05 was considered statistically significant.

## Results

A total of 563 DR patients were screened and 240 eyes of 146 treatment-naïve DR patients were enrolled. The mean age of all included patients was 56.73 ± 13.05 years, and 43.15% (63/146) of them were female. 151 eyes, including 97 PDR eyes and 54 NPDR eyes, were included in the DME group and 89 eyes, including 58 PDR eyes and 31 NPDR eyes, were included in the non-DME group. A total of 151 eyes with DME were consisted of 68 with DRT, 66 with CME, and 17 with SRD. In addition, there were 39 eyes with intact ELM/EZ, 101 eyes with disrupted ELM/EZ, and 11 eyes with ELM/EZ absent in macular central subfield in DME group. The average CMT was 359.74 ± 130.43 μm in DME group and 194.57 ± 32.13 μm in non-DME group. The average number of HRF was 18.04 ± 14.85 in DME group and 1.66 ± 2.10 in non-DME group.

**Figure 1:**
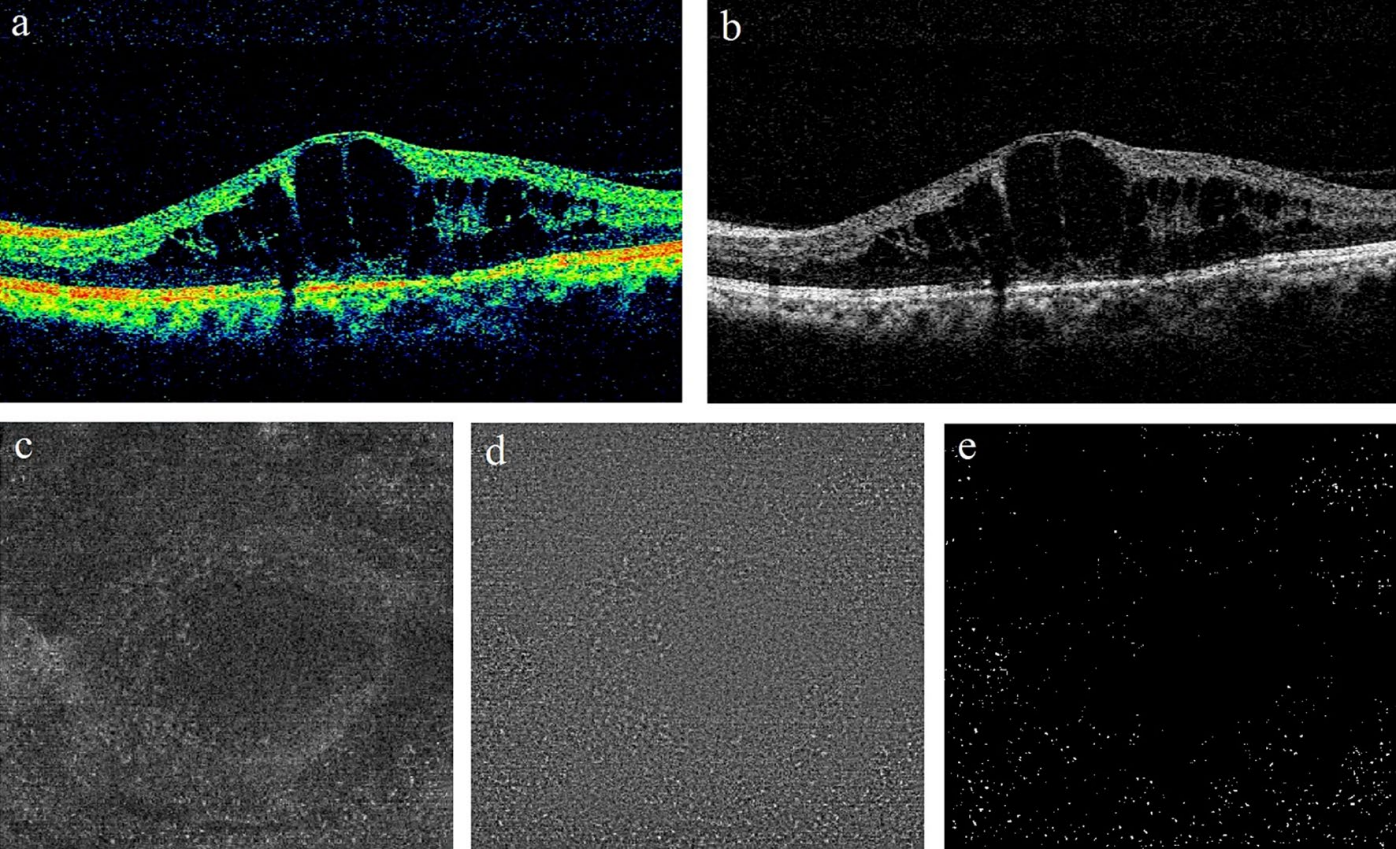
A representative example of DME. (**a,b**) B-scan of spectral domain OCT showing intraretinal fluid, with cystoid macular edema. (**c**) The en face image of customized OCT slabs showing MLCs (white dots) in macular angiography 6 × 6 mm scan by Cirrus HD-OCT 5000. (**d**) Background-flattened MLC layer by using ImageJ software. Noise reduction was performed on (**c**) to remove background irregularities and vascular artifacts. (**e**) Signal enhancement of MLCs on (**d**) to improve cell identification by using ImageJ software. *DME* diabetic macular edema, *OCT* optical coherence tomography, *MLCs* macrophage-like cells, *HD* high definition.

**Figure 2:**
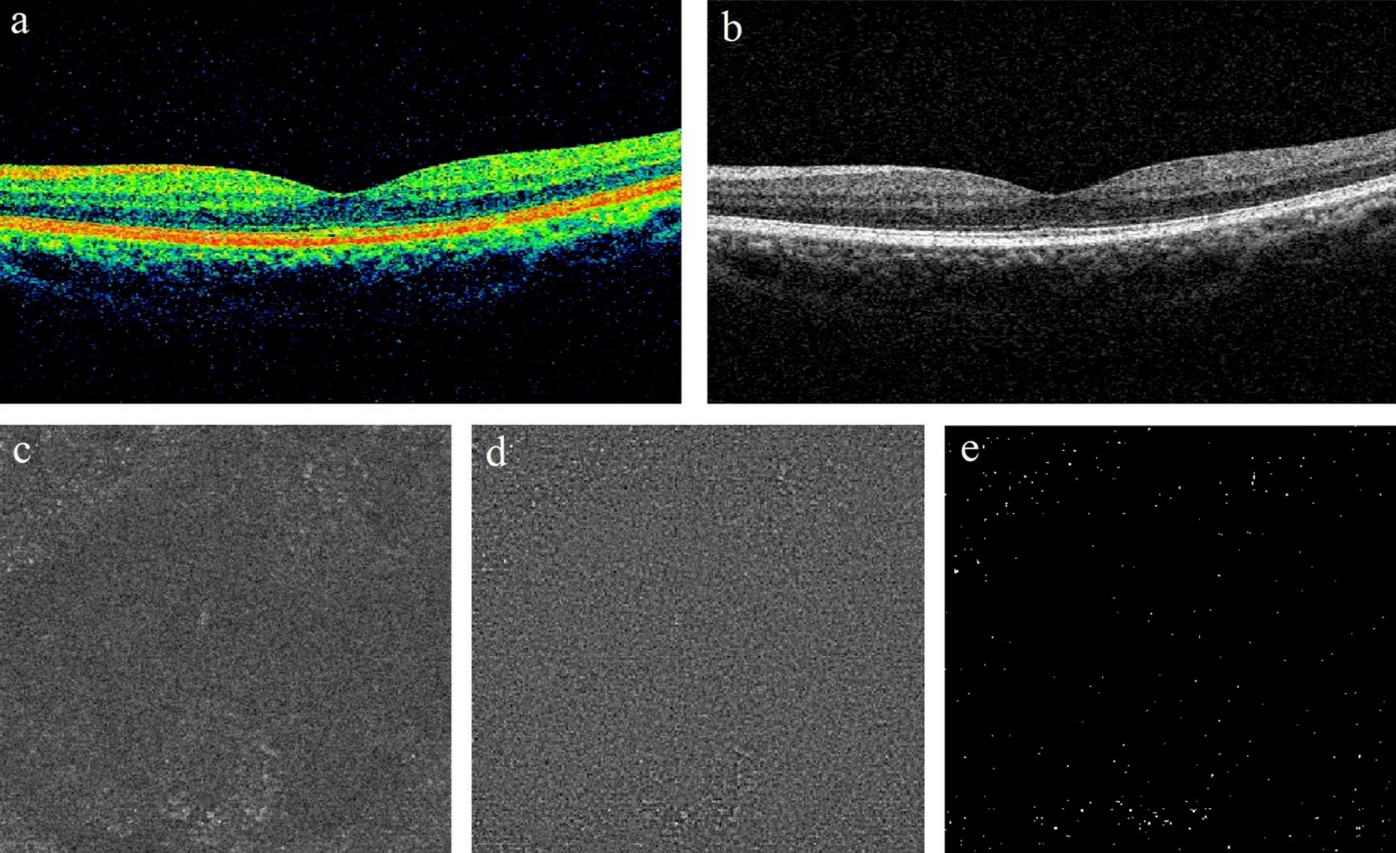
A representative example of non-DME DR eye. (**a,b**) B-scan of spectral domain OCT showing no apparent structural abnormalities in the macular region. (**c**) The en face image of customized OCT slabs showing MLCs (white dots) in macular angiography 6 × 6 mm scan by Cirrus HD-OCT 5000. (**d**) Background-flattened MLC layer by using ImageJ software. Noise reduction was performed on (**c**) to remove background irregularities and vascular artifacts. (**e**) Signal enhancement of MLCs on (**d**) to improve cell identification by using ImageJ software. *DME* diabetic macular edema, *OCT* optical coherence tomography, *MLCs* macrophage-like cells, *HD* high definition.

**Figure 3:**
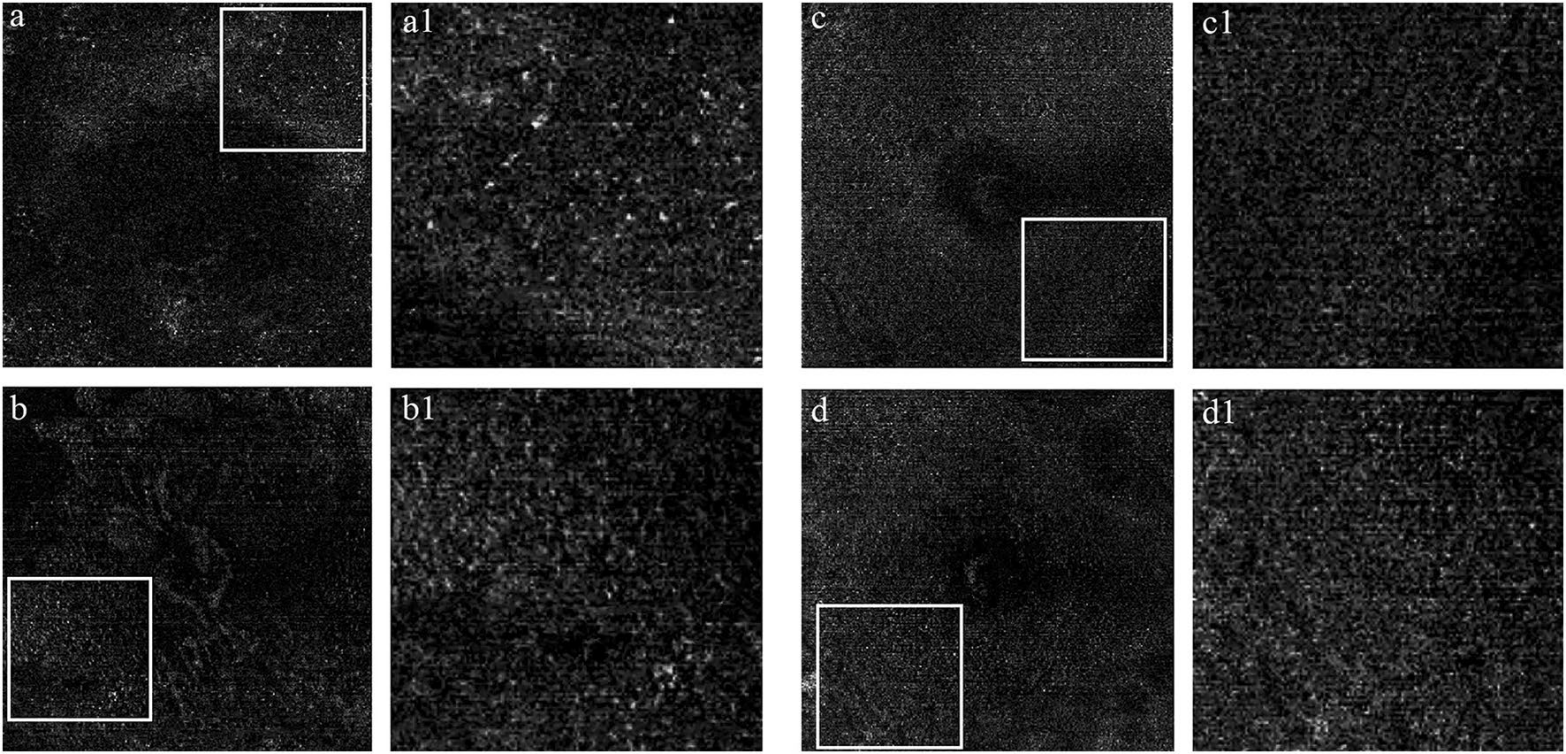
Representative examples of morphological characteristics of MLCs in DME group (**a,b**) and non-DME group (**c,d**). The 3-μm en face OCT slabs showing MLCs at macular region. The regions with relatively higher MLCs density in 6 × 6 mm macular region were identified and enlarged respectively on the right side of the original figures (**a1,b1,c1,d1**). Compared with non-DME group, the morphology of the MLCs appeared to be lager and fuller with more protrusions in DME group. *DME* diabetic macular edema, *OCT* optical coherence tomography, *MLCs* macrophage-like cells, *HD* high definition.

The retinal and vascular parameters in DME group and non-DME group were listed in Table 1. The number of macular MLCs in DME group was significantly larger comparing to non-DME group (781.79 ± 337.45 vs. 590.07 ± 314.15, p < 0.001) and so was the density of macular MLCs (21.72 ± 9.37 vs. 16.3 ± 8.73 cells/mm^2^, p < 0.001). The mean number and density of macular MLC in DME group was 1.32 times and 1.33 times higher than that in non-DME group. Figures 1 and 2 show representative examples of the B-scan OCT images, en face OCT images, MLCs identified in ImageJ in DME and non-DME group. Furthermore, we also found the morphology of MLCs in DME group appeared to be lager and fuller with more protrusions than those in non-DME group (Fig. 3).

**Table 1:** The comparisons of MLCs, retinal and vascular parameters between DME eyes group and non-DME eyes group, PDR eyes and NPDR eyes in DME group and non-DME group.

	Groups		p	DME		p	Non-DME		p
	DME	non-DME		PDR	NPDR		PDR	NPDR	
Subjects, n (%)	151 (62.9)	89 (37.1)	–	97 (64.2)	54 (35.8)	–	58 (65.2)	31 (34.8)	–
Macular VD in SCP									
Central macular region	6.75 ± 3.75	4.10 ± 3.29	< 0.001	6.52 ± 3.67	7.30 ± 3.91	0.227	3.67 ± 3.25	5.19 ± 3.22	0.047
Inner layer macular region	13.93 ± 3.46	12.00 ± 3.76	< 0.001	13.76 ± 3.19	14.18 ± 3.92	0.479	11.30 ± 3.48	13.40 ± 3.87	0.015
Outer layer macular region	15.05 ± 2.99	13.66 ± 3.33	0.001	14.81 ± 2.88	15.46 ± 3.15	0.208	13.06 ± 3.29	14.91 ± 3.14	0.016
Overall macular region	14.58 ± 2.98	13.03 ± 3.34	< 0.001	14.34 ± 2.82	14.96 ± 3.26	0.231	12.37 ± 3.25	14.32 ± 3.21	0.012
Central macular thickness (um)	359.74 ± 130.43	194.57 ± 32.13	< 0.001	368.07 ± 135.86	348.27 ± 119.45	0.382	186.69 ± 29.99	208.64 ± 30.50	0.002
Macular MLC count	781.79 ± 337.45	590.07 ± 314.15	< 0.001	759.11 ± 328.72	837.85 ± 346.30	0.176	534.47 ± 267.49	675.00 ± 376.16	0.027
Area (mm^2^)	36.00 ± 0.04	36.00 ± 0.09	0.703	36.00 ± 0.04	36.00 ± 0.03	0.420	36.00 ± 0.07	36.01 ± 0.12	0.656
Macular MLC density (cells/mm^2^)	21.72 ± 9.37	16.39 ± 8.73	< 0.001	21.09 ± 9.13	23.27 ± 9.62	0.177	16.42 ± 3.16	18.75 ± 10.45	0.026
Retinal HRF	18.04 ± 14.85	1.66 ± 2.10	< 0.001	18.51 ± 15.19	17.20 ± 14.18	0.609	1.79 ± 2.02	1.42 ± 2.23	0.430
ELM/EZ integrity, n (%)			< 0.001			0.002			–
Intact	39 (25.8)	89 (100.0)		16 (16.5)	23 (42.6)		58	31	
Disrupted	101 (66.9)	0		73 (75.3)	28 (51.9)		0	0	
Absent	11 (7.3)	0		8 (8.2)	3 (5.5)		0	0	
Leakage types in FFA, n (%)			–			0.495			
Focal leakage	39 (25.8)	0		22 (22.7)	17 (31.5)				
Diffuse leakage	48 (31.8)	0		32 (33.0)	16 (29.6)				
Mixed leakage	64 (42.4)	0		43 (44.3)	21 (38.9)				

*DME* diabetic macular edema, *NPDR* non-proliferative diabetic retinopathy, *PDR* proliferative diabetic retinopathy, *VD* vessel density, *SCP* superficial capillary plexus, *MLC* macrophage-like cell, *HRF* hyperreflective foci, *ELM* external limiting membrane, *EZ* ellipsoid zone, *FFA* fundus fluorescein angiography.

**Figure 4:**
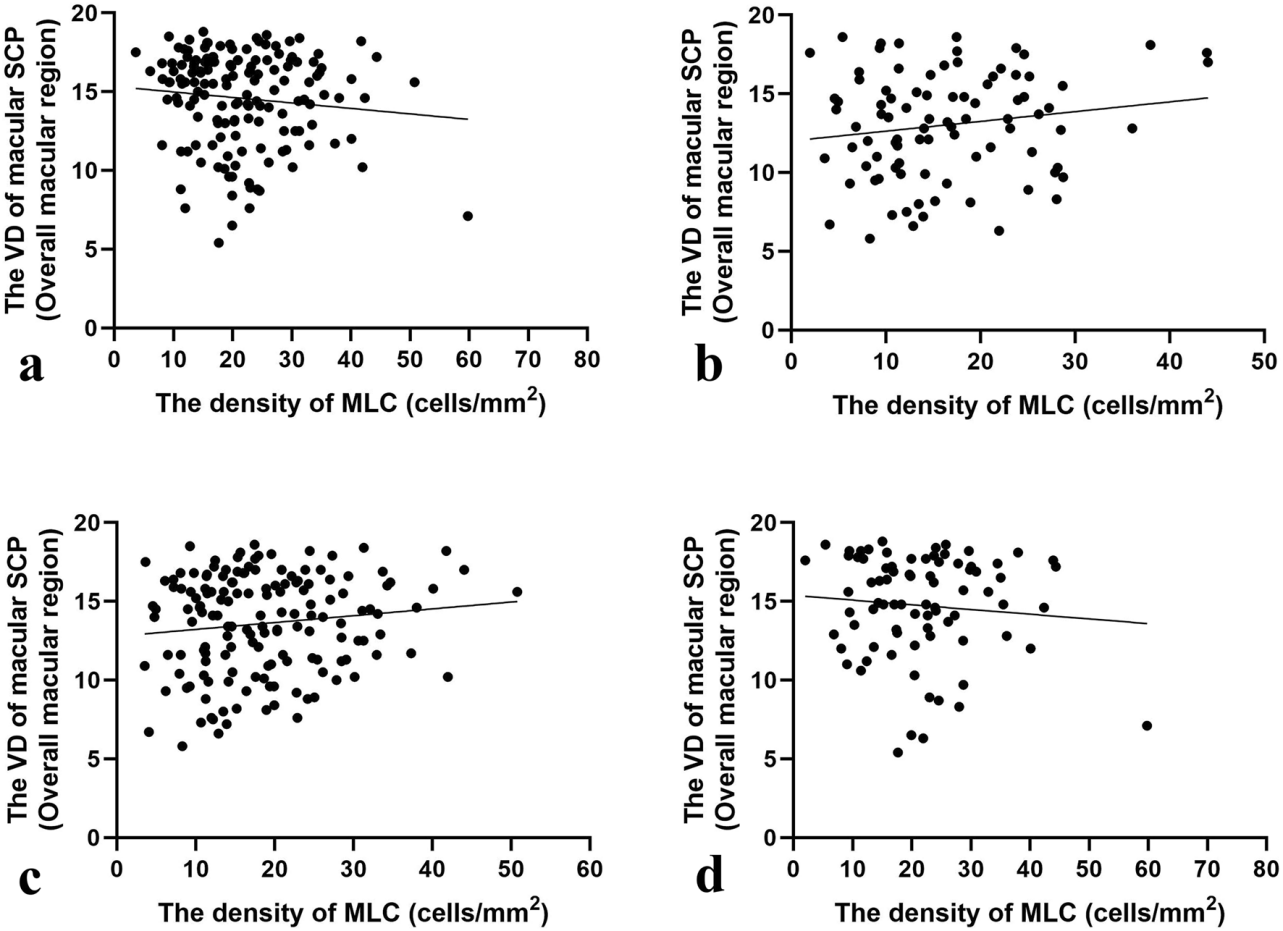
Scatter plots of Pearson’s correlation analysis between the density of MLC and the VD of macular SCP. (**a**) Scatter plots of Pearson’s correlation analysis demonstrating the association between the density of MLC and the VD in overall macular region of SCP in DME group. (**b**) Scatter plots of Pearson’s correlation analysis demonstrating the association between the density of MLC and the VD in overall macular region of SCP in non-DME group. (**c**) Scatter plots of Pearson’s correlation analysis demonstrating the association between the density of MLC and the VD in overall macular region of SCP in PDR eyes of DME and non-DME groups. (**d**) Scatter plots of Pearson’s correlation analysis demonstrating the association between the density of MLC and the VD in overall macular region of SCP in NPDR eyes of DME and non-DME groups. *MLCs* macrophage-like cells, *VD* vessel density, *SCP* superficial capillary plexus, *DME* diabetic macular edema, *PDR* proliferative diabetic retinopathy, *NPDR* non-proliferative diabetic retinopathy.

In DME group, the number and density of MLCs in NPDR were 1.10 times of that in PDR patients, but there was no statistical difference between NPDR and PDR eyes in DME group (Table 1). However, in non-DME group, NPDR eyes had higher number of MLCs comparing to PDR eyes (675.00 ± 376.16 vs. 534.47 ± 267.49, p = 0.027) and higher density of MLCs (18.75 ± 10.45 vs. 16.42 ± 3.16 cells/mm^2^, p = 0.026) (Table 1). Although NPDR eyes had greater vessel density of SCP in overall macular region comparing to PDR eyes in non-DME group (14.32 ± 3.21 vs. 12.37 ± 3.25, p = 0.016), Pearson's correlation analysis showed that the density of MLC had no correlation with the vessel density of SCP in overall macular region in DME group (p = 0.181) nor in non-DME group (p = 0.129) (Fig. 4a,b). No correlation was found between the density of MLC and the vessel density of SCP in overall macular region in PDR eyes (p = 0.120) nor in the NPDR eyes (p = 0.412) (Fig. 4c,d).

In DME group, according to the DME leakage features of FFA, the number of MLCs was 220.23 ± 112.32 in focal leakage subgroup, 184.35 ± 102.74 in diffuse leakage subgroup, and 154.30 ± 95.45 in mixed leakage subgroup, which showed statistically difference (p = 0.008). However, the number of MLCs was 186.21 ± 112.76 in DRT subgroup, 179.88 ± 103.85 in CME subgroup, and 155.76 ± 83.49 in SRD subgroup with no significant difference between them (p = 0.578). Neither was the difference significant in number of MLCs among subgroups with different ELM/EZ integrity (161.13 ± 89.08 in intact ELM/EZ subgroup, 188.93 ± 115.33 in disrupted ELM/EZ subgroup, and 165.09 ± 52.52 in ELM/EZ absent subgroup, p = 0.345). Pearson’s correlation analysis showed that the number and density of MLC both had no correlation with the number of HRF in overall macular region in DME group (p = 0.124 and 0.201, respectively).

## Discussion

Numerous animal models and in vitro studies have demonstrated that microglia reactivity and elevated levels of pro-inflammatory cytokines and chemokines are important hallmarks in DR pathologies^17–22^. Although MLCs, like microglia and hyalocytes, have been proved to be related to inflammation and involved in the pathogenesis of DME, retinal vein occlusion (RVO), epiretinal membrane (ERM), proliferative vitreoretinopathy (PVR), and PDR ex vivo, the visualization of MLCs in vivo was challenging. Recently, Castanos et al. and Hammer et al. used OCT to image MLCs located at the vitreoretinal interface^5,6^. This method avoids the influence of lipid exudation and ensure the accuracy of detecting MLCs. In our study, we discovered the number and density of MLCs at macular region as a novel inflammation biomarker were significantly higher in DR patients with DME than those without DME. This finding was supportive to the theory of inflammation in DME. And, to our knowledge, this is the first study to observe the change of MLCs in DME using OCTA.

Although there was no statistical difference in the number and density of MLCs between PDR and NPDR eyes in DME group, we observed the statistically significant differences in non-DME group between PDR and NPDR eyes, and NPDR eyes tended to present more MLCs than PDR in DME group. We speculated that greater ischemia in PDR eyes might reduce the number and aggregation of MLCs which rely upon the glycolysis of the endothelial cells^7,23^.

Previous study found that MLCs were larger and plumper in PDR and they postulate this was related to the breakdown of the blood-retinal barrier (BRB) followed by more activation of microglia and aggregation of circulating macrophage^23,24^. But in our study, MLC number and density were comparable between PDR and NPDR eyes in DME group. In this group, SCP vessel density was also comparable between PDR and NPDR eyes (Table 1). Conversely, in non-DME group, NPDR eyes which had higher SCP vessel density had higher number and density of MLCs comparing to PDR eyes. This finding raised a possibility that the number and density of MLCs may be related to the vessel density of SCP at macular region. Previous studies reported a similar finding that the MLCs were less likely to be found in the non-perfused areas in PDR and RVO.

Interestingly, the macular MLC number and density did not correlate with SCP vessel density at macular region in both DME and non-DME group as we first expected. There are several possible explanations of this phenomenon. First, the SCP vessel density may not be reliable due to many reasons such as segmentation difficulties when there is edema or exudate. We did not analyze the deep capillary plexus (DCP) because we think projection artifact can be a challenge which may interfere our quantification of vessel density in DCP. Although comparing to DCP, vessel density in SCP is more reliable, it’s accuracy can still be affected by many reasons. Secondly, there may be other factors associated with the MLC number and density. This was supported by the fact that MLC was activated and aggregated in diseases without retinal vascular changes such as ERM, PVR or open-angle glaucoma. We speculated that inflammation, oxidative stress or neurodegeneration may be also involved in the activation of MLCs^25,26^. Thirdly, relatively small sample may lack the power to discover the relationship between MLCs and vessel density at macular region. Fourthly, we only did the 6 × 6 mm OCTA scan but MLCs may not only relate to vessel density at macular region but also the entire retina. Previous study has reported that activation of MLCs may be generalized even in the occlusion-free region of RVO. Inflammation generated by local retina could spread to the adjacent area or even the entire retina^13^.

In our study, DME group had greater CMT and number of MLCs comparing to non-DME group. This finding was similar to the result of a study by Zeng et al., which found that RVO eyes with a higher density of MLCs tended to have greater macular thickness^13^. But whether macular edema was the origin or the result of MLCs activation was still unknown. Ex vivo studies have found that inflammation can activate microglia, activated microglia could migrate to alter the function of BRB, release more inflammatory cytokines and aggravate inflammation in a vicious circle.

In DME subgroup analysis, we found that MLCs were significantly different among different DME leakage types in FFA, with the most significant difference between focal leakage and mixed leakage (p = 0.006). Nevertheless, no correlations were found between the number of MLCs and the number of HRF. Although HRF were postulated to be related to inflammation component in DME pathogenesis, there have always been controversies. There were several hypotheses about the origin of HRF, it could be precursors of hard exudates^27^, migrating RPE^28^, degenerated photoreceptor cells^29^, or activated microglia^30^. Previous investigations have found that HRF in the inner retinal layers responded well to therapy which is in accordance with microglial cell behavior, but the HRF in the outer retinal layers responded poorly to therapy which suggest that at least some HRF might be precursors of hard exudates or migrating RPE^31^. Our study found that there was no correlation between MLCs and the number of HRF in DME, supporting the theory that HRF might be an OCT sign which sharing common appearance but having different origins.

DME and non-DME patients included in this study were all treatment-naïve DR patients, thus effectively excluding confounding factors resulting from interferences such as intravitreal anti-vascular endothelial growth factor (VEGF) injection or laser therapy. But there are still some limitations of this study. First, the area outside the 6 × 6 mm macular scan area were not evaluated. With the development of wide-field OCTA we may have more information about MLCs. Secondly, there is no unified and standard protocol to quantify MLCs, therefore we followed the methods in previous publication to measure the number and density of MLCs. There also might be systemic error in binarization processing with ImageJ. We believe that these limitations could be resolved with development of OCTA technology in the future.

In summary, the number and density of MLCs at macular region were significantly higher with larger and fuller morphology in DR patients with DME than those without DME. PDR eyes had fewer MLCs than NPDR eyes for DR eyes without DME.

## Supplementary Information

Supplementary Information.

## Data Availability

The data used to support the findings of this study are available from the corresponding author upon request.
