# Supplementary material for: Elevated number and density of macrophage-like cell as a novel inflammation biomarker in diabetic macular edema
Source: Sci Rep. 2023 Mar 31;13:5320. doi: 10.1038/s41598-023-32455-1 (PMC10066327; doi:10.1038/s41598-023-32455-1)
Supplement: Supplementary file 1 — Supplementary Information. [file 41598_2023_32455_MOESM1_ESM.docx]

According to the methods for spot detection in fluorescence microscopy by Janice X et al., a semiautomated binarization process implemented as a FIJI macro was used to identify and isolate MLCs from the MLC layer OCT en face slab.

(Ong, J. X., Nesper, P. L., Fawzi, A. A., Wang, J. M. & Lavine, J. A. Macrophage-Like Cell Density Is Increased in Proliferative Diabetic Retinopathy Characterized by Optical Coherence Tomography Angiography. Invest Ophthalmol Vis Sci 62, 2, doi:10.1167/iovs.62.10.2 (2021))

run("Close All");

imageID = getString("Enter patient ID", "default");

directory = // Insert filename here // + imageID + "/";

ilmID = "AVG_Registered ilm.tif";

rnflID = "AVG_Registered rnfl-oct.tif";

open(directory+ilmID);

run("32-bit");

open(directory+ilmID);

run("32-bit");

run("Invert");

run("Gaussian Blur...", "sigma=5");

saveAs("PNG", directory+"GaussInv.png");

imageCalculator("Add create", ilmID,"GaussInv.png");

selectWindow("Result of "+ilmID);

setOption("ScaleConversions", true);

run("8-bit");

saveAs("PNG", directory+"flat.png");

run("Subtract Background...", "rolling=5");

saveAs("PNG", directory+"flat+RB5_preVesselDel.png");

open(directory+"flat+RB5_preVesselDel.png");

selectWindow("flat+RB5_preVesselDel.png");

setTool("Paintbrush Tool");

setForegroundColor(255,255,255);

run("Paintbrush Tool Options...", "brush=2");

waitForUser("Use the brush tool to mark out areas of vessel

artifact to be REMOVED. Click OK when finished.");

selectWindow("flat+RB5_preVesselDel.png");

imageCalculator("Subtract create",

"flat+RB5_preVesselDel.png","flat+RB5_preVesselDel-1.png");

selectWindow("Result of flat+RB5_preVesselDel.png");

run("Multiply...", "value=255.000");

saveAs("PNG", directory+"VesselSeeds.png");

run("Morphological Reconstruction", "marker=[VesselSeeds.png]

mask=flat+RB5_preVesselDel.png type=[By Dilation]

connectivity=4");

imageCalculator("Subtract create", "flat+RB5_preVesselDel.png",

"VesselSeeds-rec");

selectWindow("Result of flat+RB5_preVesselDel.png");

saveAs("PNG", directory+"flat+RB5.png");

run("32-bit");

open(directory+rnflID);

run("8-bit");

run("Invert");

run("Gaussian Blur...", "sigma=2");

saveAs("PNG", directory+"RNFL_GaussInv.png");

selectWindow("GaussInv.png");

run("8-bit");

imageCalculator("Average create",

"GaussInv.png","RNFL_GaussInv.png");

selectWindow("Result of GaussInv.png");

saveAs("PNG", directory+"ILM+RNFL_GaussInv.png");

run("32-bit");

selectWindow("flat+RB5.png");

run("32-bit");

imageCalculator("Multiply create",

"flat+RB5.png","ILM+RNFL_GaussInv.png");

selectWindow("Result of flat+RB5.png");

saveAs("PNG", directory+"flat+RB5+comp.png");

run("8-bit");

run("Subtract Background...", "rolling=10");

saveAs("PNG", directory+"flat+RB5+comp+RB10.png");

selectWindow("GaussInv.png");

close();

selectWindow("RNFL_GaussInv.png");

close();

selectWindow("ILM+RNFL_GaussInv.png");

close();

selectWindow("VesselSeeds.png");

close();

selectWindow("VesselSeeds-rec");

close();

selectWindow("flat+RB5_preVesselDel.png");

close();

selectWindow("flat+RB5_preVesselDel-1.png");

close();

selectWindow("flat+RB5+comp+RB10.png");

run("Auto Threshold", "method=MaxEntropy white");

saveAs("PNG", directory+"autoSeeded_raw.png");

selectWindow("autoSeeded_raw.png");

selectWindow(ilmID);

run("8-bit");

imageCalculator("Add", ilmID, "autoSeeded_raw.png");

selectWindow(ilmID);

setTool("Paintbrush Tool");

setForegroundColor(0,0,0);

run("Paintbrush Tool Options...", "brush=5");

waitForUser("Use the brush tool to remove UNWANTED automatic

seeds. Click OK when finished.");

selectWindow(ilmID);

run("8-bit");

open(directory+ilmID);

run("8-bit");

imageCalculator("Subtract create", ilmID, "AVG_Registered ilm-

1.tif");

selectWindow("Result of "+ilmID);

run("Multiply...", "value=255.000");

saveAs("PNG", directory+"autoSeeded.png");

selectWindow("autoSeeded_raw.png");

close();

selectWindow(ilmID);

close();

selectWindow("AVG_Registered ilm-1.tif");

close();

// open(directory+"autoSeeded.png");

selectWindow("flat+RB5.png");

run("8-bit");

run("Morphological Reconstruction", "marker=[autoSeeded.png]

mask=flat+RB5.png type=[By Dilation] connectivity=4");

selectWindow("autoSeeded-rec");

saveAs("PNG", directory+"auto_MorphoDil.png");

selectWindow("autoSeeded.png");

close();

selectWindow("auto_MorphoDil.png");

run("Auto Threshold", "method=MaxEntropy white");

saveAs("PNG", directory+"Macs_auto.png");

open(directory+"auto_MorphoDil.png");

imageCalculator("Subtract create", "flat+RB5.png",

"auto_MorphoDil.png");

selectWindow("Result of flat+RB5.png");

saveAs("PNG", directory+"flat+RB5+Macs_auto-removed.png");

selectWindow("auto_MorphoDil.png");

close();

// selectWindow("flat+RB5+Macs_auto-removed.png");

open(directory+ilmID);

run("RGB Color");

selectWindow("Macs_auto.png");

run("8-bit");

run("Red");

imageCalculator("Transparent-zero create", ilmID,

"Macs_auto.png");

selectWindow("Result of "+ilmID);

setTool("Paintbrush Tool");

setForegroundColor(255,255,255);

run("Paintbrush Tool Options...", "brush=1");

waitForUser("Use the brush tool to add manual seeds for any

missed macrophages. Click OK when finished.");

// open(directory+"flat+RB5+Macs_auto-removed.png");

// run("8-bit");

selectWindow("Result of "+ilmID);

imageCalculator("Subtract", "Result of "+ilmID, ilmID);

selectWindow("Macs_auto.png");

run("Grays");

selectWindow("Result of "+ilmID);

run("8-bit");

imageCalculator("Subtract", "Result of "+ilmID,

"Macs_auto.png");

selectWindow("Result of "+ilmID);

// imageCalculator("Subtract create", "flat+RB5+Macs_autoremoved.

png", "flat+RB5+Macs_auto-removed-1.png");

// selectWindow("Result of flat+RB5+Macs_auto-removed.png");

run("Multiply...", "value=255.000");

saveAs("PNG", directory+"manualSeeded.png");

selectWindow("flat+RB5+Macs_auto-removed.png");

run("Morphological Reconstruction", "marker=manualSeeded.png

mask=flat+RB5+Macs_auto-removed.png type=[By Dilation]

connectivity=4");

selectWindow("manualSeeded-rec");

saveAs("PNG", directory+"manual_MorphoDil.png");

run("Auto Local Threshold", "method=Bernsen radius=150

parameter_1=0 parameter_2=0 white");

saveAs("PNG", directory+"Macs_manual.png");

imageCalculator("Add create",

"Macs_auto.png","Macs_manual.png");

selectWindow("Result of Macs_auto.png");

run("Set Scale...", "distance=304 known=3 unit=mm");

saveAs("PNG", directory+"Macs_final.png");

selectWindow("flat+RB5.png");

close();
